# Effects of C1-INH Treatment on Neurobehavioral Sequelae and Late Seizures After Traumatic Brain Injury in a Mouse Model of Controlled Cortical Impact

**DOI:** 10.1089/neur.2022.0011

**Published:** 2023-03-09

**Authors:** Min Chen, Quang M. Tieng, Jiaxin Du, Stephen R. Edwards, Dhiraj Maskey, Emil Peshtenski, David Reutens

**Affiliations:** Centre for Advanced Imaging, The University of Queensland, Brisbane, Queensland, Australia.

**Keywords:** C1-INH, epileptogenesis, neurobehavioral sequelae, traumatic brain injury

## Abstract

C1 human-derived C1 esterase inhibitor (C1-INH) is a U.S. Food and Drig Administration–approved drug with anti-inflammatory actions. In the present study, we investigated the therapeutic effects of C1-INH on acute and chronic neurobehavioral outcomes and on seizures in the chronic stage in a mouse traumatic brain injury (TBI) model. Adult male CD1 mice were subjected to controlled cortical impact and randomly allocated to receive C1-INH or vehicle solution 1 h post-TBI. Effects of C1-INH treatment on inflammatory responses and brain damage after TBI were examined using the Cytometric Bead Array, C5a enzyme-linked immunosorbent assay, Fluoro-Jade C staining, and Nissl staining. Neurobehavioral outcomes after TBI were assessed with modified neurological severity scores, the rotarod and open field tests, and the active place avoidance task. Video-electroencephalographic monitoring was performed in the 15th and 16th weeks after TBI to document epileptic seizures. We found that C1-INH treatment reduced TNFα expression and alleviated brain damage. Treatment with C1-INH improved neurological functions, increased locomotor activity, alleviated anxiety-like behavior, and exhibited an effect on seizures in the chronic stage after TBI. These findings suggest that C1-INH has beneficial effects on the treatment of TBI.

## Introduction

Globally, traumatic brain injury (TBI) affects 69 million persons and causes 8.1 million years of life lived in disability each year.^1,2^ Most moderate and severe TBI survivors have chronic neurological, cognitive, and behavioral sequelae and increased rates of psychiatric illness such as anxiety.

TBI triggers a series of changes, including neuroinflammation, blood–brain barrier (BBB) breakdown, alteration of the epigenetic landscape, and reorganization of neural circuitry, which increase seizure susceptibility and the risk of spontaneous recurrent seizures. Post-traumatic epilepsy (PTE) is a condition characterized by at least two spontaneous recurrent seizures occurring as a result of TBI.^3^ It affects 17–27% of patients after severe closed TBI and 27–53% of patients after penetrating TBI or depressed skull fracture.^4^ Although supportive and rehabilitative care after TBI has improved, there is a gap in effective treatments targeting the pathophysiology underlying secondary damage to improve functional recovery and prevent PTE.

TBI triggers multiple sequalae, including various inflammatory mechanisms such as activation of resident microglial cells, complement system activation, BBB damage, platelet recruitment and activation, and peripheral immune cell infiltration.^5–7^ Although the precise interplay between inflammatory and neuronal mechanisms is still not fully understood, evidence is mounting that inflammatory mediators play an important role in brain damage and epileptogenesis.^8,9^ C1-INH is a member of the serpin family of protease inhibitors, which inactivates proteases involved in the activation of the complement system, contact-kinin system, and fibrinolytic/coagulation system. It also has anti-inflammatory functions independent of protease inhibition, such as suppressing leukocyte transmigration across the endothelium.^10^

CI-INH is U.S. Food and Drug Administration (FDA) approved for the treatment of hereditary angioedema. Its therapeutic potential has been investigated in rodent models of TBI. Longhi and colleagues evaluated the effects of 15-IU C1-INH treatment on motor function using a composite neuroscore, weekly from 1 to 4 weeks post-injury, and assessed cognitive function at 4 weeks post-injury in a mouse controlled cortical impact (CCI) model.^11,12^ C1-INH attenuated motor deficits, cognitive dysfunction, and reduced contusion volume when given 10 min after TBI. However, delayed administration of C1-INH at 1 h post-injury only reduced motor deficits, but had no effects on cognitive and histological outcomes, suggesting a narrow therapeutic window for C1-INH treatment after TBI.

More recent studies, however, demonstrated that there is a longer time window of C-INH treatment for brain injury. For instance, 15 IU of C1-INH administered 1 h post-injury ameliorated brain injury, protected the BBB, attenuated inflammatory responses, and inhibited thrombus formation in a mouse cortical cryolesion model.^13^ Treatment with 15 IU of C1-INH 1 h after stroke also reduced infarct volumes and improved clinical scores in mice.^14^ C1-INH treatment accelerated recovery of body weight, attenuated anxiety-like behavior, and reduced the level of caspase-3 in a rat status epilepticus model.^15^

**FIG. 1.**
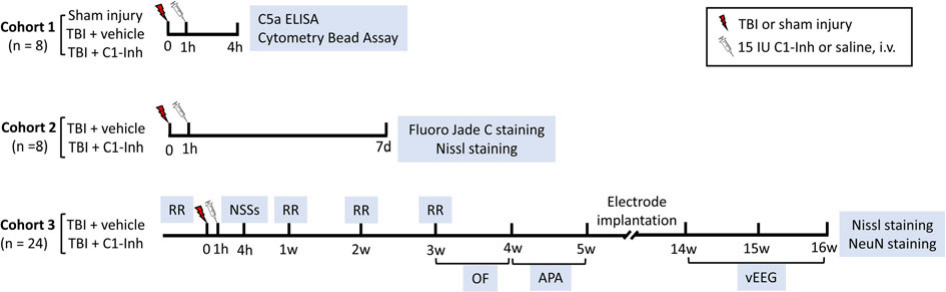
Schematic representation of timeline for experimental procedures. One cohort of animals was euthanized for C5a ELISA and Cytometric Bead Array at 4 h post-injury, and a separate cohort of animals was perfused for FJC and Nissl staining at 7 days post-TBI. In the third cohort of animals, treatment effects on neurobehavioral outcomes, post-traumatic epilepsy, and brain damage in the chronic stage were examined. TBI, traumatic brain injury; ELISA, enzyme-linked immunosorbent assay; FJC, Fluoro-Jade C staining; NSSs, neurological severity scores; RR, rotarod; OF, open field; APA, active place avoidance; vEEG, video-electroencephalography.

C1-INH ameliorated brain injury, protected the BBB, and attenuated inflammatory responses,^13,14^ which all play critical roles in the development of PTE.^8,9^ However, the effect of C1-INH treatment on the development of epilepsy after TBI remains to be tested. The present study evaluated the effects of C1-INH administered at 1 h post-TBI on neurobehavioral outcomes, the risk of developing epilepsy, and its severity in a mouse model of CCI. Treatment effects on neurobehavioral outcomes, including acute neurological, motor and locomotor function, anxiety-related behavior, spatial learning ability, and PTE, were examined (Fig. 1). This study is the first to examine the effect of early C1-INH treatment on epileptic seizures in the chronic stage post-TBI.

## Methods

Experimental procedures were approved by the University of Queensland Animal Ethics Committee (Approval No.: CAI/300/17) and the Animal Care and Use Review Office of the US Army Medical Research and Development Command. All behavioral tests were performed by operators blinded to operations and treatments.

### Controlled cortical impact model and C1-INH treatment

Adult outbred CD1 mice (Envigo, Indianapolis, IN) 9–10 weeks of age were subjected to a severe unilateral cortical contusion as previously described.^16^ Mice were anesthetized with a mixture of tiletamine/zolazepam (50 mg/kg; Virbac, Carros, France) and xylazine (20 mg/kg; Troy Laboratories, Glendenning, NSW, Australia). Craniotomy was performed by removing the skullcap 4 mm to the left of the sagittal suture and centered between the bregma and lambda. A CCI injury was delivered by compressing the cortex to a depth of 2.0 mm at a velocity of 5.0 m/s and 100-ms duration with a beveled steel tip 3 mm in diameter (TBI-0310; Precision Systems and Instrumentation, Fairfax, VA). Sham-operated mice received the craniotomy procedure without cortical impact. One hour after brain injury, mice were randomly allocated to receive a single intravenous injection of human plasma-derived C1-INH (15.0 IU of Berinert^®^; CSL Behring GmbH, Marburg, Germany) or equal volumes of vehicle control (isotonic saline).

### C5a enzyme-linked immunosorbent assay

Ipsilateral hemispheres of the injury site were snap-frozen in liquid nitrogen and stored at −80°C before being ground to a fine powder using a mortar and pestle on dry ice. Brain tissues were homogenized and centrifuged, and supernatants were collected and stored as described previously.^17^ C5a expression levels were determined using enzyme-linked immunosorbent assay (ELISA; #DY2150; R&D Systems, Minneapolis, MN). The measured concentration of C5a was normalized to a protein concentration, which was measured by a bicinchoninic acid protein assay (#23227; ThermoFisherScientific, Waltham, MA).

### Cytometric Bead Array

The Cytometric Bead Array (#552364; BD Biosciences, San Jose, CA) was used to measure interleukin (IL)-12p70, tumor necrosis factor alpha (TNFα), interferon-gamma (IFNγ), monocyte chemoattractant protein 1 (MCP-1), IL-10, and IL-6 in the ipsilateral hemisphere on an LSRII flow cytometer (BD Biosciences). Results were analyzed using FCAP array (version 3.0) software. Calculated cytokine concentrations were normalized to the total protein content of each sample.

### Fluoro-Jade C staining

Brain sections (20-μm cryostat brain sections) were stained with Fluoro-Jade C to detect neuronal death as described previously.^18^ Briefly, brain slides were incubated in 100% ethanol (3 min), 70% ethanol (1 min), distilled water (1 min), and then a solution containing 0.0004%, Fluoro-Jade C (Merck Millipore, Darmstadt, Germany), and 0.1% acetic acid for 30 min. Sections were washed three times in distilled water, then dried overnight before being immersed in xylene (3 × 2 min) and mounted with DPX neutral mounting medium (Sigma-Aldrich, St. Louis, MO). Three coronal brain sections (200 μm between slices, −1.2 and −2.2 mm from bregma) from each mouse were imaged with a fluorescence microscope (BX63; Olympus, Tokyo, Japan). Fluoro-Jade C–positive cells were counted using the CellSens Count and Measure Solution module (Olympus) in the perilesional region of the ipsilateral hemisphere by an investigator blind to the treatment.

### Nissl staining

Brain sections were washed in deionized water (10–15 sec) before passing through 70% ethanol (3 min), 100% ethanol (2 × 3 min), and xylene (2 × 3 min). Sections were rehydrated by passing back through 100% ethanol (2 × 1 min), 70% ethanol (1 min), and deionized water (10–15 sec). Sections were then incubated in 0.1% cresyl violet (Sigma-Aldrich) acetate solution for 5–10 min before being differentiated in 70% ethanol (30 sec), dehydrated in 100% ethanol (2 × 1 min), cleared in xylene (2 × 3 min), and mounted with DPX^®^ neutral mounting medium. Images were acquired using an Olympus microscope (BX63; Olympus) and were analyzed using ImageJ software (National Institutes of Health, Bethesda, MD).^19^ In the brain sections from mice euthanized at 7 days after TBI, the lesion was identified as brain regions with darkly stained, shrunken cells at lower density than contralaterally and brown discoloration corresponding to hemorrhage. The lesion area was measured and compared between vehicle- and C1-INH-treated groups. In each mouse euthanized at 16 weeks post-TBI, the percent area of tissue loss in the ipsilateral hemisphere was quantified: [(area of contralateral hemisphere-area of ipsilateral hemisphere) / (area of contralateral hemisphere)] × 100.

### Immunostaining

Antigen retrieval was performed by immersing sections in 10 mM of citrate buffer (pH = 6.0) at 90°C for 10 min using a pressure cooker. Brain sections were blocked in 1% bovine serum albumin in phosphate-buffered saline containing 1% Triton X-100 for 30 min, then incubated with neuronal marker chicken anti-neuronal nuclei (NeuN; 1:5000; ABN91; Merck Millipore) for ∼56 h and Alexa Fluor^TM^ 555 goat anti-chicken (1:1000; A11039; ThermoFisherScientific) overnight at room temperature. Sections were counterstained with 4′,6-diamidino-2-phenylindole (DAPI) dye (1:1000; Sigma-Aldrich) for 5 min to stain the nuclei and mounted using DABCO (Sigma-Aldrich). Cells labeled with NeuN and DAPI in the ipsilateral cortex near the lesion region were measured using the Colocalization pipeline of CellProfiler Image Analysis software (Broad Institute, Cambridge, MA).

### Neurological severity scores

Neurological severity scores (NSSs) measure the presence of reflexes and ability to perform motor tasks, including beam walking, beam balancing, and spontaneous locomotion, with 10 tasks, as previously described.^20,21^ One point was given for failing to perform each of the tasks: 1) presence of mono- or hemiparesis; 2) ability to walk on a 3-cm-wide beam (30 cm long in 2 min); 3) ability to walk on a 2-cm-wide beam (30 cm long in 2 min); 4) ability to walk on a 1-cm-wide beam (30 cm long in 2 min); 5) ability to balance on a 1-cm-wide beam for at least 10 sec; 6) ability to balance on a round stick (0.5-cm diameter) for at least 10 sec, 7) ability to exit a 30-cm-diameter circle within 2 min; 8) seeking behavior; 9) ability to walk straight; and 10) startle behavior. NSSs were assessed at 4 h after CCI injury. All behavioral studies were performed in a blinded manner

### Rotarod

Mice were subjected to three training trials at a rotational speed of 4–16 rpm in 60 sec (Ugo Basile, Comerio, Italy) the day before the injury, followed by three testing trials with accelerating rotational speed from 4 to 40 in 180 sec with a minimum 15-min intertrial interval. Mice underwent three trials, and the average time to fall from the rotating cylinder was recorded as latency at 1, 2, and 3 weeks after injury.

### Open field

The open field test was conducted using a square arena (27.3 × 27.3 × 20.3 cm) equipped with infrared beams (Med Associates, Inc., Fairfax, VT). In each experiment, the mouse was placed in the center of the arena, and the movement was recorded over 20 min. Cumulative movement duration and average velocity were recorded, along with the entrance to the central zone, with analyses performed using EthoVision XT software (version 13; Noldus Information Technology, Wageningen, The Netherlands).

### Active place avoidance

The active place avoidance apparatus (Bio-Signal Group, Acton, MA) consisted of an elevated arena (77 cm in diameter), with a grid floor fenced with a transparent circular boundary (32 cm high). The day before the initial experiments, mice were placed in the arena, which rotated counterclockwise (1 rpm) for 5 min.^22^ Over 5 consecutive days, mice were subjected to 10-min sessions in the rotating arena (1 rpm counterclockwise), with visual cues displayed on each wall. An overhead camera tracked the mice, and a mild electric shock (500 ms, 60 Hz, 0.6 mA at 1.5-sec intervals) was delivered when they entered the shock zone, a 60-degree region of the grid kept constant in relation to room coordinates. Recorded tracks of mice were analyzed offline using Track Analysis software (Bio-Signal Group, Acton, MA).

### Video-electroencephalography recording

Electrode implantation and video-electroencephalography (vEEG) recording were performed as previously described.^16,18,23^ Briefly, one recording electrode (1-mm-diameter stainless steel screw) was inserted into the skull rostral to the midline of the craniotomy. Another recording electrode was positioned contralateral to a region corresponding to the center of the craniotomy. A reference electrode was placed above the right frontal cortex, and a ground electrode was inserted into the occipital bone over the cerebellum. All electrodes were connected to a plastic pedestal (MS 363; Plastics One, Inc., Roanoke, VA) and cemented onto the skull with dental acrylic.

vEEG data were acquired at 15 and 16 weeks post-injury. EEG baseline was recorded from non-injured animals (*n* = 3) for 4 weeks, and no electrographic seizures were detected in these mice. EEG data were filtered (high-pass filter: 1 Hz; low-pass filter: 30 Hz) and analyzed by investigators blinded to the experimental condition and treatment. Our laboratory developed and validated the MATLAB software (The MathWorks, Inc., Natick, MA) to detect seizures for EEG data from two recording electrodes in a similar configuration to that used in this study.^24^

Electrographic seizures were first automatically detected by an investigator using the purpose-written MATLAB code, using multiple features, including permutation entropy, phase coherence, negative logarithm of adaptive correlation integral, and Power Spectrum Coherence Ratio, to optimally exploit both linear and non-linear aspects of the EEG signal, as described previously.^24^ Then, another investigator, blinded to the treatment group, visually inspected synchronized vEEG data to verify all the electrographic seizures and exclude artifacts caused, for example by chewing, grooming, or scratching. An electrographic seizure was defined as a high-amplitude (>2 × baseline), rhythmic discharge or spike and wave pattern with a clear onset, offset, and temporal evolution in frequency and morphology, lasting >10 sec. Only events with electrographic changes accompanied by freezing behavior, head nodding and tail extension, or with forelimb clonus were included. No generalized convulsive seizures, with rearing and falling, were observed.

### Statistical analysis

Statistical analysis was performed using GraphPad Prism software (GraphPad Software Inc., La Jolla, CA).

Comparison of NSSs, rotarod, and active place avoidance between vehicle- and C1-INH-treated mice at different time points was performed using two-way analysis of variance (ANOVA). Data for vehicle- and C1-INH-treated mice at a single time point were compared using Student's *t*-test. All values are expressed as mean ± standard error of the mean. The threshold for statistical significance was set at *p* ≤ 0.05 for all analyses.

## Results

### C1-INH reduced inflammation and neurodegeneration

**FIG. 2.**
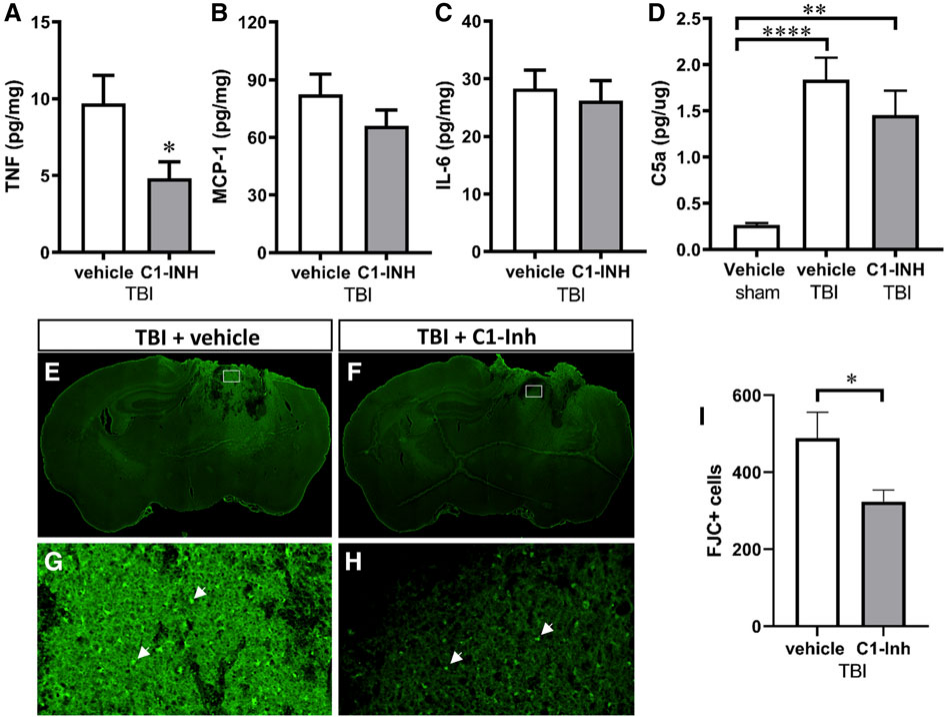
Effect of C1-INH treatment 1 h after TBI exposure on inflammation and neurodegeneration at 2 days after TBI. (**A–C**) Compared to vehicle treatment, C1-INH treatment significantly reduced TNFα (*n* = 8, unpaired Student's *t*-test; *p < 0.05) at 2 days post-TBI, but did not affect the expression of MCP-1 and IL-6. (**D**) TBI dramatically increased C5a level compared to sham injury (*n* = 8, one-way ANOVA; vehicle TBI vs. vehicle sham, ****p < 0.0001; C1-INH TBI vs. vehicle sham, **p < 0.01), but there was no significant difference between vehicle treatment and C1-INH treatment. Representative Fluoro-Jade C staining in vehicle-treated (**E,G**) and C1-INH-treated (**F,H**) brain sections. (**I**) C1-INH treatment reduced Fluoro-Jade C–positive cells in the perilesional region of the ipsilateral hemisphere at 2 days after TBI (*n* = 8; unpaired Student's *t*-test; *p < 0.05). FJC, Fluoro-Jade C staining.

First, we examined the effects of C1-INH treatment on the presence of cytokines and chemokines, including TNFα, MCP-1, IL-6, IL-12p70, IFNγ, and IL-10, in the ipsilateral hemisphere at 4 h after TBI using a mouse inflammation cytometric bead assay. Levels of IL-12p70, IFNγ, and IL-10 in brain tissue were too low to be analyzed accurately. In sham mice, TNFα, MCP-1, and IL-6 levels were very low (mean ± standard error = 0.25 ± 0.10, 2.88 ± 1.00, and 0.23 ± 0.06 pg/mg, respectively) and were out of range of the standard control curve of the Cytometric Bead Assay. C1-INH treatment decreased TNFα expression compared with vehicle treatment (Fig. 2A; *p* < 0.05), but had no significant effects on MCP-1 and IL-6 levels (Fig. 2B,C). We also assessed the effect of C1-INH treatment on complement activation using C5a ELISA. TBI increased C5a expression, but C1-INH did not affect C5a expression significantly (Fig. 2D).

**FIG. 3.**
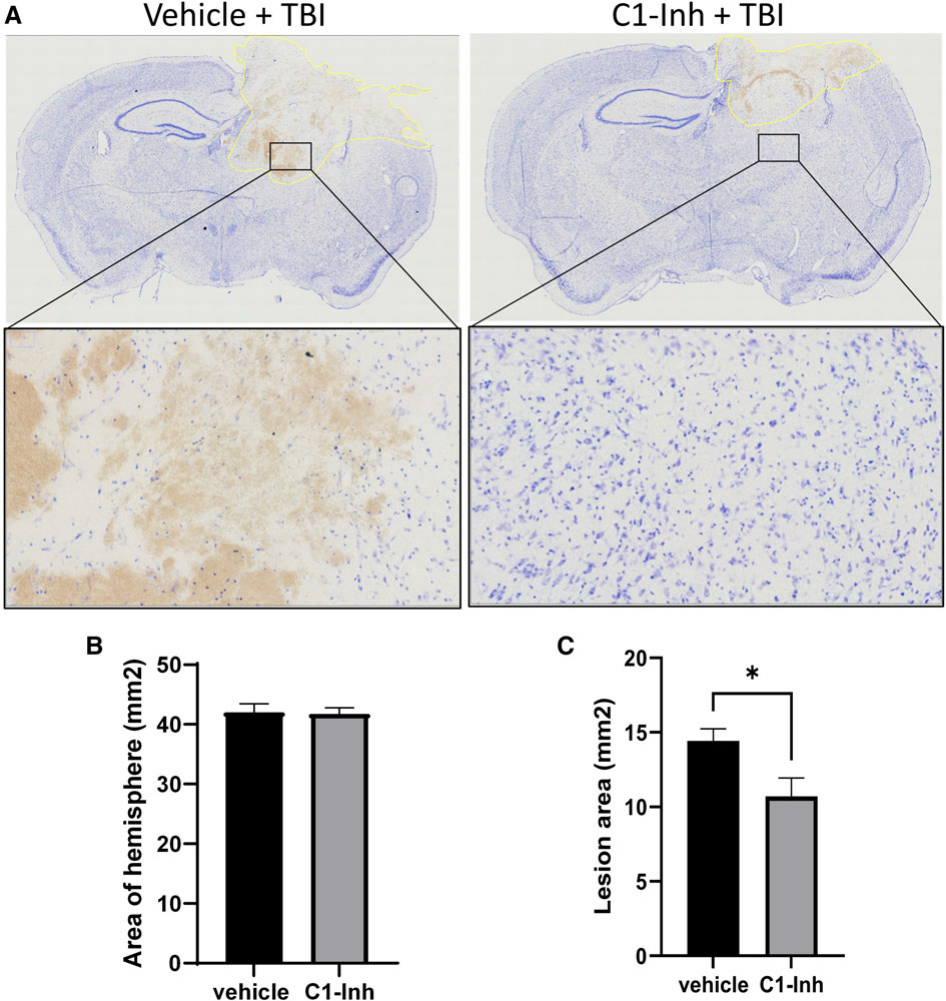
Effects of C1-INH on brain lesion at 7 days after TBI. (**A**) Representative Nissl staining images of vehicle-treated TBI mouse and C1-INH-treated TBI mouse. Lesion regions are indicated by solid yellow lines. Enlarged images show reduced neuronal density and brown plaques in a region of interest in the thalamus. C1-INH treatment reduced lesion area (**C**), but did not affect the area of the ipsilateral hemisphere (**B**). TBI, traumatic brain injury.

We examined neuronal protective effects 7 days after TBI using Fluoro-Jade C staining and Nissl staining. Fluoro-Jade C staining detects all degenerating neurons regardless of specific insult or mechanism of cell death. Fluoro-Jade C–positive cells were mainly observed in the lesioned cortex and ipsilateral thalamus (Fig. 2E–H). C1-INH treatment significantly reduced Fluoro-Jade C–positive cells compared to vehicle treatment (Fig. 2I; *p* < 0.05). Nissl staining showed darkly stained, shrunken cells at lower density than contralaterally, areas of brown discoloration caused by hemorrhage, and distorted microstructure in lesional cortical, thalamic, and hippocampal regions (Fig. 3A). C1-INH treatment reduced lesion area, but did not significantly affect the area of the ipsilateral hemisphere (Fig. 3B,C).

### C1-INH improved acute neurological functions after traumatic brain injury

**FIG. 4.**
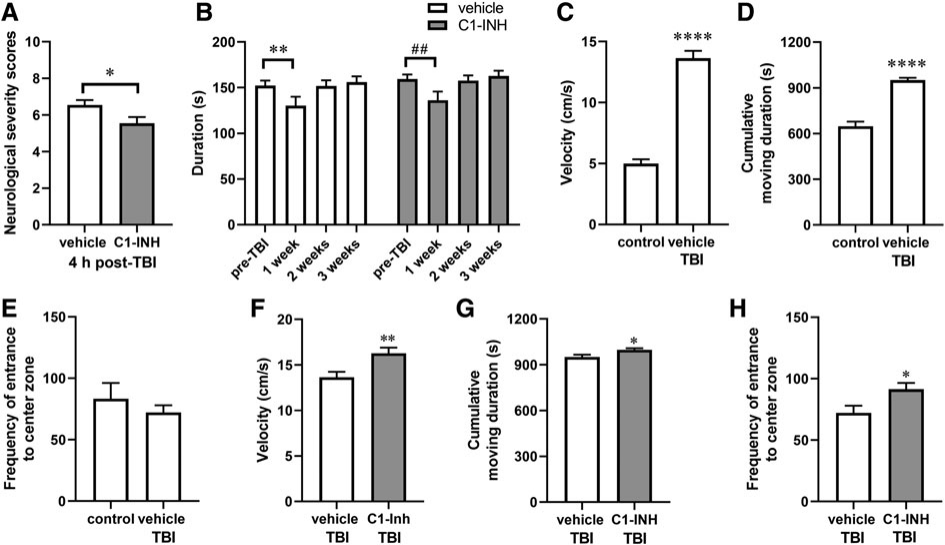
Effects of C1-INH treatment on neurological severity scores (NSSs), rotarod performance, and open field test after controlled cortical impact injury. (**A**) C1-INH treatment significantly reduced NSSs compared to vehicle treatment at 4 h post-injury (*n* = 24; unpaired Student's *t*-test; *p < 0.05). (**B**) In rotarod tests, latency to fall was significantly lower at 1 week post-injury compared to pre-TBI baseline and recovered at 2 and 3 weeks after TBI in both vehicle- and C1-INH-treated mice (*n* = 24; two-way ANOVA, Dunnett's multiple comparisons test; *p < 0.05 compared to pre-TBI in the vehicle-treated group; ^#^*p* < 0.05 compared to pre-TBI in the CI-INH-treated group). There was no significant difference between vehicle and C1-INH treatments (Bonferroni's multiple comparisons test; *p* > 0.05 at all time points). (**C–E**) TBI increased velocity and cumulative duration of movement (control, *n* = 10; C1-INH, *n* = 24; unpaired Student's *t*-test; *p < 0.05), but not frequency of entrance to the central zone compared to the normal control group 4 weeks post-injury. (**F–H**) C1-INH treatment increased velocity, cumulative duration of movement, and frequency of entrance to the central zone at 4 weeks after TBI (*n* = 24; unpaired Student's *t*-test; *p < 0.05). ANOVA, analysis of variance; TBI, traumatic brain injury.

We examined the effect of C1-INH treatment on functional neurological status, including motor ability, balance, and sensorimotor reflexes, by assessing NSSs at 4 h, 1 day, 2 days, and 3 days after TBI. In a pilot study, we tested the NSSs in pre-TBI mice (*n* = 24), and all mice performed all 10 tasks successfully without training. C1-INH treatment significantly reduced NSSs at 4 h after TBI (Fig. 4A; *p* < 0.05), indicating that C1-INH treatment improved acute neurological function. Because of the rapid recovery of neurological function, evidenced by the low NSSs (score <1) in both treatment groups from 1 day after TBI, C1-INH- and vehicle-treated mice did not differ significantly at 1–3 days after TBI (data not shown).

### C1-INH increased locomotor function and reduced anxiety-like behavior

We investigated the effects of C1-INH on motor function, including grip strength, balance, and motor coordination, using rotarod tests at 1, 2, and 3 weeks after TBI. Compared to pre-TBI baseline, fall latencies 1 week after TBI were significantly lower in both vehicle-treated (Fig. 4B; *p* < 0.01) and C1-INH-treated animals (Fig. 4B; *p* < 0.01). No significant differences were observed between C1-INH and vehicle treatment 1 week after TBI, indicating that C1-INH treatment did not affect the gross motor function assessed by rotarod tests (Fig. 4B).

Open field tests were conducted 3 weeks after TBI to determine locomotor activity and exploratory behavior over a 20-min testing period. Compared to normal controls, TBI increased movement velocity (Fig. 4C; *p* < 0.0001) and cumulative movement duration (Fig. 4D; *p* < 0.0001), but did not change the frequency of entrance to the central zone (Fig. 4E). Movement velocity (Fig. 4F; *p* < 0.01) and cumulative movement duration (Fig. 4G; *p* < 0.05) were significantly higher in the C1-INH treatment group compared to the vehicle-treated group. The number of entries into the central area of the arena and cumulative time spent in this area were used as indicators of anxiety-like behavior. C1-INH treatment significantly increased the frequency of entry into the central zone (Fig. 4H; *p* < 0.05). Collectively, these findings indicate that C1-INH treatment increased locomotor activity levels and reduced anxiety-related behavior.

### C1-INH had no effects on spatial learning ability

**FIG. 5.**
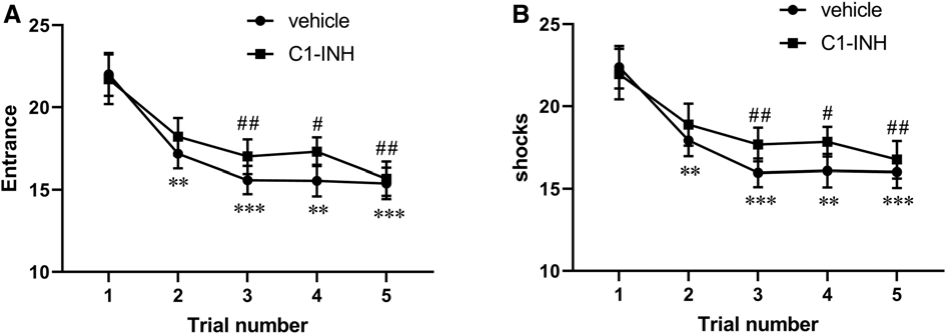
Effects of C1-INH on performance in active place avoidance trials over 5 days. Compared to the first trial, the entrance number (**A**) and shock number (**B**) were lower from the second trial onward in the vehicle-treated group (repeated-measures two-way ANOVA; *n* = 24; **p < 0.01) and from the third trial onward in the C1-INH-treated group (*n* = 24; ^##^*p* < 0.01). There was no significant difference between vehicle and C1-INH treatment. ANOVA, analysis of variance.

A previous study in the CCI mouse model demonstrated that TBI mice made significantly more mistakes (increased number of entries into the shock zone) than sham-injured mice and failed to demonstrate improvements in performance in five trials of active place avoidance task over 5 consecutive days.^22,25^ We assessed the effect of C1-INH treatment on active place avoidance tasks using the same experimental settings (10-min sessions over 5 consecutive days) at 5 weeks after TBI. The number of entrances to the shock zone and the number of shocks delivered when mice entered the shock zone were compared over a 5-day testing period. Compared to day 1, entrance number and shock number were significantly lower from day 2 in vehicle-treated mice (Fig. 5A; *p* < 0.01 and Fig. 5B; *p* < 0.001) and from day 3 in C1-INH-treated mice (Fig. 5A; *p* < 0.01 and Fig. 5B; *p* < 0.01). However, there were no significant differences in entrance numbers and shock numbers between C1-INH- and vehicle-treated mice in any trials, indicating that C1-INH treatment did not improve spatial learning ability.

### C1-INH exhibited effects on late seizures

**FIG. 6.**
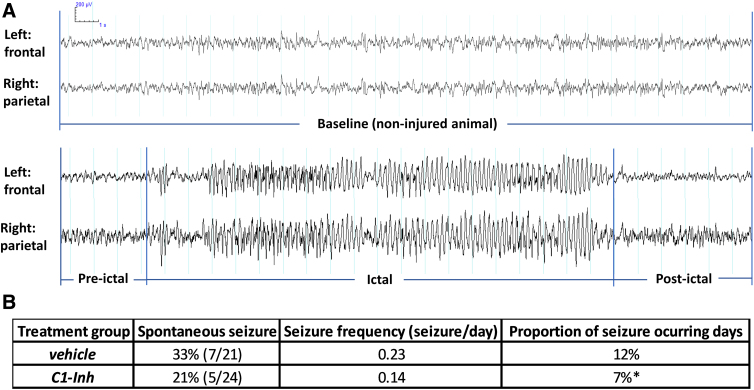
Effects of C1-INH treatment on late seizures. (**A**) EEG traces from a non-injured mouse showing baseline background and a spontaneous seizure with high-amplitude repetitive spikes and spike waves. (**B**) Spontaneous seizures were observed in 7 of 21 (33%) vehicle-treated mice and 5 of 24 (21%) C1-INH-treated mice during the period of vEEG recording. Seizure frequency in vehicle-treated animals was higher than in C1-INH-treated animals (0.23 ± 0.47 vs. 0.14 ± 0.47 seizures/day), but this difference was not statistically significant (unpaired Student's *t*-test; *p* > 0.05). The proportion of seizure occurring days during vEEG monitoring was significantly lower in C1-INH-treated animals (7%) than in vehicle-treated animals (12%; Fisher's exact test, *p* < 0.05). vEEG, video-encephalography.

Before vEEG recording, 2 vehicle-treated mice were culled because of animal welfare considerations, 1 because of a wound caused by scratching and 1 because of hypothermia and loss of appetite. One vehicle-treated mouse was excluded from data analysis because of artifact from connection issues during EEG recording. Spontaneous seizures were observed in a higher proportion of vehicle-treated mice (7 of 21; 33%) compared to C1-INH-treated mice (5 of 24; 21%), during the period of recording, 15 and 16 weeks after TBI, but this difference was not statistically significant (*p* > 0.05, Fisher's exact test; Fig. 6B), possibly attributable to the overall low occurrence of PTE. Seizure clusters (at least 4 seizures/day) occurred in 3 vehicle-treated mice and 1 C1-INH-treated mouse. Seizure frequency was calculated as the number of seizures per animal for each day of monitoring. Average seizure frequency was higher in vehicle-treated animals (0.23 seizures/day) compared to C1-INH-treated animals (0.14 seizures/day), but the difference was not statistically significant. The proportion of days of vEEG monitoring in which seizures occurred was significantly lower in C1-INH-treated animals (7%) than in vehicle-treated animals (12%; Fisher's exact test, *p* < 0.05).

### C1-INH reduced brain damage

**FIG. 7.**
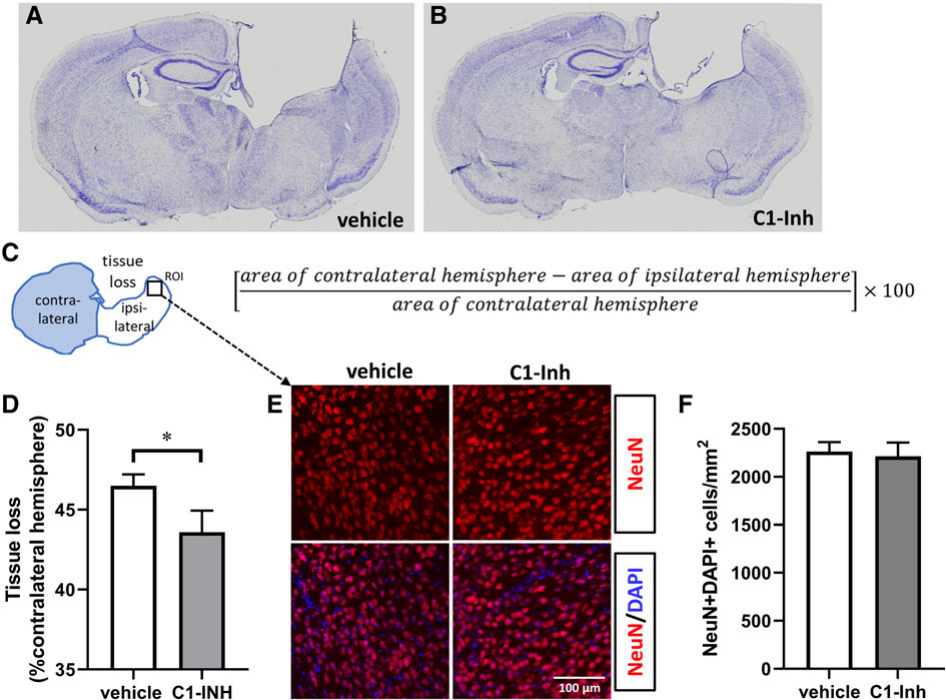
Effects of C1-INH on brain lesion at 16 weeks after TBI. Representative Nissl staining (**A,B**) and NeuN staining (**E**) in vehicle- and C1-INH-treated brain sections. (**C**) Method for quantification of Nissl staining results and region of interest (ROI) for NeuN data analysis. (**D**) C1-INH treatment significantly reduced lesion size (vehicle, *n* = 25; C1-INH, *n* = 12, unpaired Student's *t*-test; *p < 0.01). (E) NeuN/DAPI-positive cells in the cortex near the lesion area were comparable in C1-INH and vehicle groups (vehicle, *n* = 10; C1-INH, *n* = 11, unpaired Student's *t*-test; *p* > 0.05). DAPI, 4′,6-diamidino-2-phenylindole; NeuN, neuronal nuclei.

We measured area of tissue loss at the injury site 16 weeks after TBI on the Nissl-stained sections. Neuronal density in the cortex adjacent to area of tissue loss at that time point was assessed using NeuN staining. Compared to vehicle control, C1-INH treatment significantly reduced the percentage area of tissue loss in the hemisphere ipsilateral to the impact site (Fig. 7D; *p* < 0.05). However, the density of NeuN/DAPI-positive cells in the remaining cortex adjacent to the injury site was comparable in vehicle- and C1-INH-treated mice (Fig. 7E,F).

## Discussion

This study showed that C1-INH treatment improved acute functional neurological status, increased locomotor activity, decreased anxiety level, and exhibited effects on late seizures after TBI. The safety profile of C1-INH from extensive clinical experience supports the possibility of repurposing C1-INH as a treatment for severe TBI to improve neurobehavioral outcomes and potentially to reduce the severity of PTE.

Given that the treatment window of C1-INH is vital from the standpoint of clinical translation, we undertook evaluation of treatment effects of C1-INH administered 1 h post-TBI. We examined the effects of 15 IU of C1-INH administered 1 h post-injury on motor functions using three different behavioral tests across the first month after TBI. We found that C1-INH treatment improved acute neurological function, evidenced by reduced NSSs, at 4 h post-injury. Gross motor functions, including balance, grip strength, and motor coordination, assessed by rotarod test, remained impaired at 1 week post-injury and C1-INH treatment did not alter rotarod performance compared to vehicle treatment. Hyperactivity and anxiety are commonly described in humans and mice after TBI.^26,27^ We found that C1-INH treatment increased general locomotor activity and increased the frequency of entrance to the central zone in the open field test, suggesting that C1-INH increased exploratory behavior and reduced anxiety-like behavior. In addition, consistent with previous studies,^11,15^ C1-INH treatment 1 h post-TBI did not affect cognitive deficits caused by brain injury.

In a rat model of status epilepticus, C1-INH treatment also increased mobility and reduced anxiety-like behaviors, but did not attenuate status epilepticus-induced deficits in hippocampal-dependent learning and memory.^15^ However, the effects of C1-INH treatment on the development of epilepsy were not tested in previous work. We found that spontaneous seizures were observed in 33% of vehicle-treated mice, and the average seizure frequency in mice that developed epilepsy was 0.23 seizures/day. Although a lower proportion of C1-INH-treated mice (21%) had seizures, and the average seizure frequency was lower (0.14 seizures/day), the differences were not statistically significant. The proportion of days of vEEG monitoring in which seizures occurred was significantly lower in C1-INH-treated mice than in vehicle-treated mice, suggesting that C1-INH administered 1 h after CCI affects characteristics of seizures in the chronic stage after TBI.

Previous studies in clinically relevant models of traumatic and ischemic brain injury suggested that C1-INH improved neurobehavioral outcomes through anti-inflammatory mechanisms, including regulating cytokines and chemokines and inhibiting the complement system.^13,14,28^ Pre-treatment with C1-INH reduced IL-2, but it did not change the levels of TNFα and S100 calcium-binding protein B in the rat weight-drop model.^28^ In a mouse cryolesion model, C1-INH treatment at 1 h post-TBI attenuated the TBI-induced inflammatory response by reducing the expression of chemokine ligand 3, chemokine ligand 2 (also known as MCP-1), TNFα, and IL-1β.^13^ We found that C1-INH treatment significantly reduced TNFα expression, but did not affect the levels of MCP-1. C1-INH is a well-known modulator of the complement system by blocking the classical and lectin pathways.

Pre-treatment with C1-INH before TBI reduced C3a levels in the rat weight-drop model, even though the change was relatively mild (∼15%).^28^ We found that C1-INH treatment did not inhibit TBI-induced expression of C5a, suggesting other anti-inflammatory functions independent of complement inhibition; this is consistent with a previous study showing that long-term depletion of C-INH using antisense oligonucleotide caused neurovascular dysfunction, neuroinflammation, and behavioral deficits mediated by activation of the Kallikrein–Kinin System in the circulation, without complement system activation.^29^

Both plasma-derived and recombinant C1-INH are used clinically. This project used plasma-derived C1-INH, which has a significantly longer plasma half-life than recombinant C1-INH. However, a recent study reported that recombinant C1-INH had a higher ability to deposit on ischemic endothelium and exhibited more potent neuroprotective effects.^30^ Recombinant C1-INH also had a wider therapeutic window of efficacy (up to 18 h) than plasma-derived C1-INH (shorter than 6 h) in a mouse stroke model.^31^ Future studies should explore the treatment window and therapeutic effect of recombinant C1-INH in models of TBI.

## Conclusion

Our findings suggest that C1-INH administered 1 h post-injury improves recovery of sensorimotor functions, increases motor activity, and reduces anxiety-like behavior. These studies also confirmed the neuroprotective and anti-inflammatory effects of plasma-derived C1-INH. C1-INH is FDA approved for the treatment of hereditary angioedema. The safety profile of C1-INH from extensive clinical experience supports the possibility of repurposing C1-INH to combat severe TBI.

## Abbreviations Used

ANOVA: analysis of variance
BBB: blood–brain barrier
CCI: controlled cortical impact
DAPI: 4′,6-diamidino-2-phenylindole
ELISA: enzyme-linked immunosorbent assay
FDA: U.S. Food and Drig Administration
IFNγ: interferon-gamma
IL: interleukin
MCP-1: monocyte chemoattractant protein 1
NeuN: neuronal nuclei
NSSs: neurological severity scores
PTE: post-traumatic epilepsy
TBI: traumatic brain injury
TNFα: tumor necrosis factor alpha
vEEG: video-electroencephalography
